# A sorbent containing pH-responsive chelating residues of aspartic and maleic acids for mitigation of toxic metal ions, cationic, and anionic dyes†

**DOI:** 10.1039/d1ra09234k

**Published:** 2022-02-17

**Authors:** Shaikh A. Ali, Shuaib A. Mubarak, Ibrahim Y. Yaagoob, Zeeshan Arshad, Mohammad A. J. Mazumder

**Affiliations:** Chemistry Department, King Fahd University of Petroleum & Minerals Dhahran 31261 Saudi Arabia shaikh@kfupm.edu.sa jafar@kfupm.edu.sa +966 13 860 4277 +966 13 860 7836; Interdisciplinary Research Center for Advanced Materials, King Fahd University of Petroleum & Minerals Dhahran 31261 Saudi Arabia

## Abstract

*t*-Butyl hydroperoxide-initiated cycloterpolymerization of diallylaminoaspartic acid hydrochloride [(CH_2_=CHCH_2_)_2_NH^+^CH(CO_2_H)CH_2_CO_2_H Cl^−^] (I), maleic acid (HO_2_CH=CHCO_2_H) (II) and cross-linker tetraallylhexane-1,6-diamine dihydrochloride [(CH_2_=CHCH_2_)_2_NH^+^(CH_2_)_6_NH^+^ (CH_2_CH=CH_2_)_2_ 2Cl^−^] (III) afforded a new pH-responsive resin (IV), loaded with four CO_2_H and a chelating motif of NH^+^⋯CO_2_^−^ in each repeating unit. The removal of cationic methylene blue (MB) (3000 ppm) at pH 7.25 and Pb(ii) (200 ppm) at pH 6 by IV at 298, 313, and 328 K followed second-order kinetics with *E*_a_ of 33.4 and 40.7 kJ mol^−1^, respectively. Both MB and Pb(ii) were removed fast, accounting for 97.7% removal of MB within 15 min at 313 K and 94% of Pb(ii) removal within 1 min. The super-adsorbent resin gave respective *q*_max_ values of 2609 mg g^−1^ and 873 mg g^−1^ for MB and Pb(ii). IV was also found to trap anionic dyes; it removed 91% Eriochrome Black T (EBT) from its 50 ppm solutions at pH 2. The resin was found to be effective in reducing priority metal contaminants (like Cr, Hg, Pb) in industrial wastewater to sub-ppb levels. The synthesis of the recyclable resin can be easily scaled up from inexpensive starting materials. The resin has been found to be better than many recently reported sorbents.

## Abstract 2

Cycloterpolymerization of diallylaminoaspartic acid hydrochloride (I), maleic acid (II) and a cross-linker (III) afforded a new pH-responsive resin (IV), loaded with four CO_2_H and a chelating motif of NH^+^⋯CO_2_^−^ in each repeating unit.

## Graphical abstract

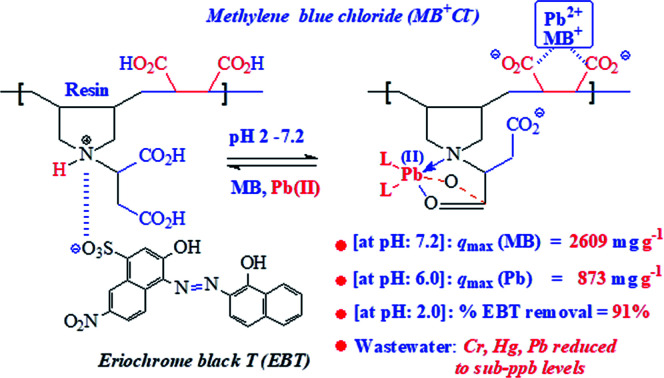

## 1. Introduction

Industrial effluents containing dyes damage the aquatic environment. Therefore, the remediation of dye-contaminated wastewater is highly significant for the protection of the environmental ecology. The textile industry uses methylene blue (MB), which is discharged into the environment *via* the effluents of textile, paper, and printing industries,^1^ thereby causing various ecological problems.^2^ Commercial annual production of dyes is ≈7 × 10^5^ tons, a considerable fraction of which is discharged directly in aqueous effluent.^3^ The dyes discharged into river streams cause enormous harm to aquatic life^4^ by reducing light penetration and photosynthesis.^5^ In addition to being carcino- and muta-genic, most dyes are not biodegradable,^6^ which makes bioremediation^7^ an inefficient process.^8^

The discharge of toxic metal ions has also added to the woes of the environment. The Chinese standard set a maximum of 0.01 mg L^−1^ for Pb in drinking water.^9^ However, the goal of the U.S. Environmental Protection Agency (EPA) is to achieve a concentration approaching zero.^10^ The water scarcity makes it imperative to remediate the wastewaters. The chelating ion-exchange resins are quite effective in removing toxic metal ions from wastewaters.^11^ The chelating ligands offer remarkable selectivity in trapping heavy metal ions in the presence of alkali and alkaline earth metal ions.

Because of simplicity and cost-effectiveness, the adsorption technique^12^ is extensively used to remove inorganic and organic contaminants from industrial wastewater.^8,13^ Among various adsorbents reported for MB removal,^14^ the relatively high cost of activated carbon limits its application, which encourages exploration for better low-cost adsorbents like biomass, clays minerals, and zeolites.^5,15^ There is demand for readily available cheap, environment friendly, and recyclable adsorbents for remediation of pollutants.

**Scheme 1:**
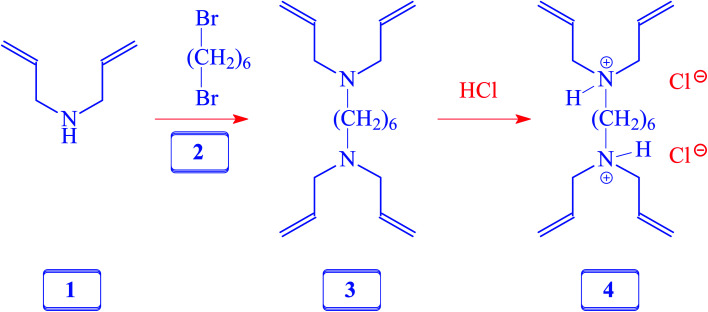
Synthesis of cross-linker 6.

**Scheme 2:**
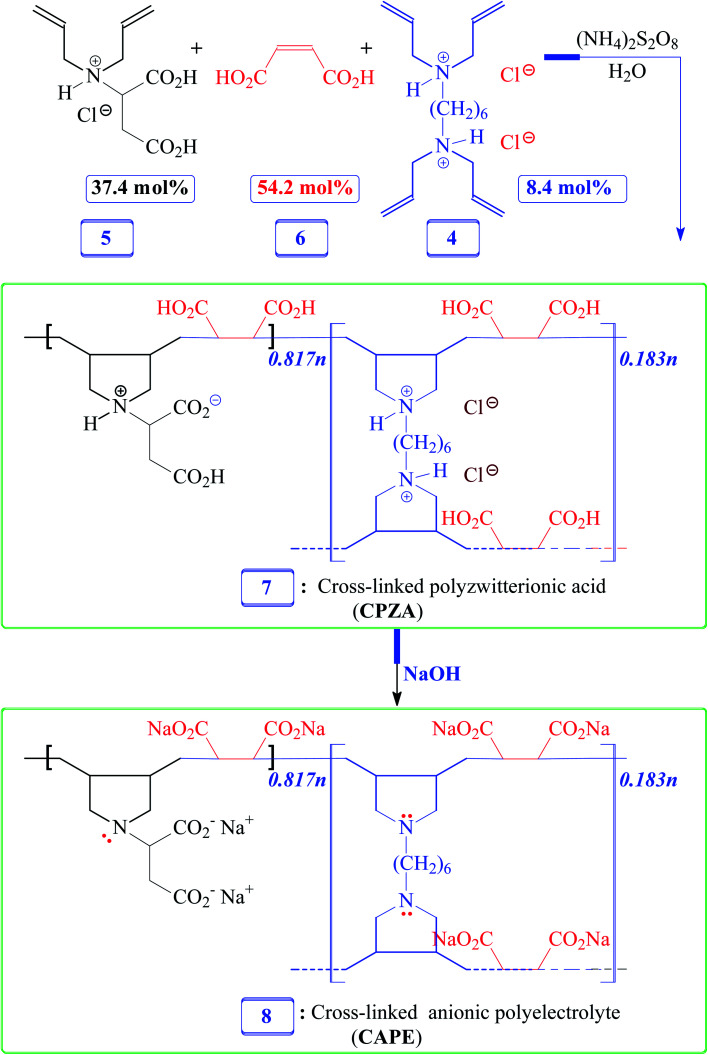
Synthesis of resins CPZA 7 and CAPE 8.

Herein, we would like to synthesize a new resin and examine its efficacy in the removal of Pb(ii), cationic dye MB, and anionic dye Eriochrome Black T (EBT) from aqueous systems. For this purpose, adsorbent cross-linked polyzwitterionic acid (CPZA) 7 has been synthesized for the first time using cyclopolymerization protocol^16–18^ involving inexpensive cross-linker 4, *N*,*N*-diallylaspartic acid hydrochloride (5) bearing the skeleton of aspartic acid [H_3_N^+^CH(CH_2_CO_2_H)_2_CO_2_^−^], and maleic acid (MA) 6 (Schemes 1 and 2). The current pH-responsive resin 7, loaded with four CO_2_H and chelating motifs of NH^+^⋯CO_2_^−^ in each repeating unit, offers the latitude for effective mitigation of cationic as well as anionic materials.

## 2. Experimental

### 2.1. Physical methods

A PerkinElmer (16F PC) spectrometer was used to record FTIR spectra. Atomic compositions were determined using a PerkinElmer instrument (Model 2400). An SDT analyzer (Q600: TA Instruments) was used to perform thermogravimetric analyses (TGA) under a flow of N_2_. Solution pHs were measured using a Sartorius pH meter. The surface morphology of the resins was examined by SEM-EDX spectroscopy. Surface area and porosimetry analyzer (Micromeritics) were used to characterize the resin using Brunauer–Emmett–Teller and Barrett–Joyner–Halenda methods. Using a UV-Vis spectrophotometer (Thermo Electron), MB concentrations were determined at pH 7 at *λ*_max_ of 665 nm. USEPA Method 6020A^19^ was utilized to determine the Pb(ii) concentrations by the Inductively Coupled Plasma-Mass Spectrometer (ICP-MS XSERIES-II), which has 0.05 ppb as the detection limit. Various Pb(ii) concentrations along with a blank were used to calibrate the ICP-MS instrument.

### 2.2. Materials

Diallylamine, dimethyl maleate, allyl chloride, 1,6-dibromohexane, *tert*-butylhydroperoxide (TBHP) were purchased from Fluka AG and used as received. MB trihydrate (C_16_H_24_ClN_3_O_3_S: molar mass 373.9), MA, and maleic acid were purchased from Sigma-Aldrich. All water used was of Milli-Q quality. ACS certified Pb(NO_3_)_2_ from Fischer Scientific Co was used to prepare a 2000 ppm Pb(ii) as a stock solution. Monomer *N*,*N*-diallylaspartic acid hydrochloride (5) was synthesized as a white solid (85%) by reacting diallylamine and dimethyl maleate using the procedure as described.^20^

### 2.3. Synthesis of *N*^1^,*N*^1^,*N*^6^,*N*^6^-tetraallylhexane-1,6-diamine 4

A solution of diallylamine 1 (33.5 g, 345 mmol) and 1,6-dibromoheane 2 (20.7 g, 85 mmol) in toluene (80 mL) was heated in a closed vessel at 115 °C for 24 h. After washing the reaction mixture with 5% NaOH (120 mL), the organic layer was dried (Na_2_SO_4_), concentrated, and distilled to obtain amine 3 as a colorless liquid (18 g, 77%, bp 0.6 mbarHg, 110 °C). ^1^H NMR and IR spectra of the amine matched with those reported^21^ for 3, which was prepared using a different route.

### 2.4. Synthesis of cross-linker 4

A solution of 3 (10 g, 36 mmol) in water (20 mL) was treated with dropwise addition of conc. HCl (37%, 8.5 g, 86 mmol) and stirred at room temperature for 30 min. The homogeneous solution was freeze-dried and the residual sticky liquid was crystallized (MeOH–ether–acetone) to obtain 4 as a white solid (11 g, 87%). Mp 138–140 °C. (Found: C, 61.6; H, 10.0; N, 7.9. C_18_H_34_Cl_2_N_2_ requires C, 61.88; H, 9.81; N, 8.02%); *v*_max_ (KBr): 3438, 3079, 2912, 2706, 2630, 2540, 1642, 1438, 1366, 1309, 1253, 1048, 997, 940, 738, and 640 cm^−1^; *δ*_H_ (D_2_O): 1.27 (4H, m), 1.62 (4H, m), 3.02 (4H, m), 3.66 (8H, d, *J* 7.05 Hz), 5.48 (8H, m), 5.80 (4H, m), (HOD: 4.65). *δ*_C_ (D_2_O): 23.83 (2C), 25.94 (2C), 52.67 (2C), 55.49 (4C), 126.36 (4C, =CH), 127.24 (4C, =CH_2_) (dioxane: 67.40 ppm).

### 2.5. Synthesis of CPZA 7

A solution of monomer 5 (17.5 mmol), maleic acid (6) (27.5 mmol), and cross-linker 4 (1.38 g, 3.95 mmol) in water (4.75 g) were prepared in an RB flask (50 mL). After purging the reaction mixture with N_2_, TBHP (600 mg) was added, and the mixture in the closed flask was stirred using a magnetic stir-bar at 75 °C for 24 h. The stir-bar became immobile; water (3 mL) was added to the gelly mixture, and the polymerization was continued at 85 °C for a further 12 h. The solidified material was soaked in water for 24 h, filtered, and washed with water. The white resin CPZA 7 was dried under vacuum at 70 °C (6.8 g, 84%). A mass of 375.5 mg of resin 7 is calculated to have 0.817 mmol of monomer (5-HCl), 1.183 mmol of maleic acid 6, and 0.183 mmol of cross-linker 4 in a 5/6/4 mol ratio of 37.4 : 54.2 : 8.4 to match the feed ratio. (Found: C, 50.8; H, 6.4; N, 4.5. Resin 7 containing monomer 5-HCl/6/4 in a mol ratio 37.4 : 54.2 : 8.4 requires C, 51.82; H, 6.23; N, 4.42%); *v*_max_ (KBr): 3566, 2937, 2526, 1731,1597, 1395, 1190, 1029, 810, and 629 cm^−1^.

### 2.6. Conversion of CPZA 7 to CAPE 8

A sample of resin 7 (1.00 g) (containing 2.18 mmol RU of 5 (–HCl), 3.15 mmol of 6, and 0.487 mmol of 4) is calculated to have 8.48 mmol protonated CO_2_H and 3.15 mmol protonated NH^+^. The sample was stirred for 2 h at room temperature in water (10 mL) containing NaOH (1.4 g, 35 mmol). The mixture, upon filtration and washing with excess methanol (remove unreacted NaOH), afforded CAPE 8 (vacuum dried at 60 °C, 1.1 g, ≈100%). A sample of CAPE 8 (450 mg) contains repeating units of the disodium salt of monomer 5 (0.817 mmol), disodium maleate (1.183 mmol), and unprotonated amine form 3 (0.183 mmol). (Found: C, 42.2; H, 4.4; N, 3.5%. Resin 8 containing disodium 5/disodium 6/3 in a mol ratio 37.4 : 54.2 : 8.4 requires C, 43.24; H, 4.22; N, 3.69%). *v*_max_ (KBr): 3484, 2933, 2858, 2803, 1660, 1582, 1399, 1298, 1208, 1151, 1108, 1029, 877, and 812 cm^−1^.

### 2.7. Ion exchange capacity (IEC)

The sample was centrifuged after stirring 100 mg of CAPE 8 with 25 mL of 0.1 M HCl for 6 h. Upon titration of a certain volume of the resin-free liquid with 0.1 M NaOH, the excess acid was calculated to determine the IEC using eqn (1):1
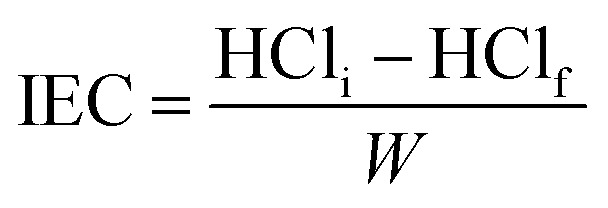
where the mass of the resin in g is represented by *W*, and the initial and final amount of HCl (mmol) are denoted by HCl_i_ and HCl_f_. The IEC was found to be 7.65 mmol g^−1^.

### 2.8. Swelling coefficient (SC)

A certain mass of the 20 to 30 mesh size resins was filled in a burette and then immersed in water. After 12 h, the volume changes were measured, and the SCs (final/initial volume ratio) was found to be 1.3 and 4.0 for resins 7 and 8, respectively. When resin 7 was covered with 1 M HCl instead of neutral H_2_O, its zwitterionic motifs were transformed to cationic motifs, giving an SC value of 2.1.

### 2.9. Adsorption of experiments

#### 2.9.1. Adsorption of MB

A magnetic stir-bar with a rotation speed of 300 rpm was used in all the adsorption experiments. In a series of vials, mixtures of CPZA 7 (50 mg) and 100–5000 ppm (*C*_o_) MB solutions (20 mL) were stirred (300 rpm) for 6 h at 298, 313, and 328 K. The pH of the solutions, was periodically checked and adjusted to 7.25 using 0.1 M NaOH. After centrifugation, the supernatant (after appropriate dilution and pH adjustment to 7) was analyzed to determine the equilibrium MB concentrations (*C*_e_) using a spectrometer at *λ* 665 nm. Several concentrations (0.1–5.0 mg L^−1^) of MB were used to construct a calibration curve.

Eqn (2) was used to calculate the adsorption capacity, *q*_e_ (mg g^−1^), where the volume, *V* of MB solution is expressed in L and *m* denotes the mass of CPZA 7 in g.2
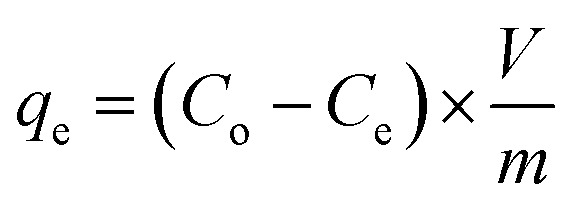


Eqn (3) expresses the percent MB-removal (*R*).3
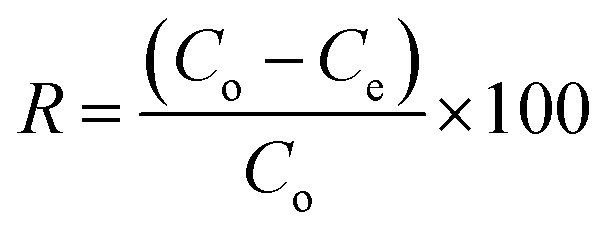


RB flask of 250 mL size containing CPZA 7 (250 mg) in water (100 − *x* mL) and *x* mL of 1.0 M NaOH was used to conduct the kinetic runs at various temperatures. Several pretrial experiments were performed to find out the value of *x* mL as ≈1.5 mL required to adjust the final pH to 7.25 subsequent to the addition of MB trihydrate in solid form (351 mg) (MW 373.9 g mol^−1^), to make the concentration of MB (MW 319.85 g mol^−1^) as 3000 ppm. After stirring the mixture using a magnetic stir-bar (300 rpm), the stirring was briefly stopped at several time intervals to collect ≈0.2 mL of the supernatant, which was used after appropriate dilution to determine the MB concentrations at pH 7. Eqn (4) was to determine the adsorption capacity, *q*_*t*_ (mg g^−1^) at various time *t*.4
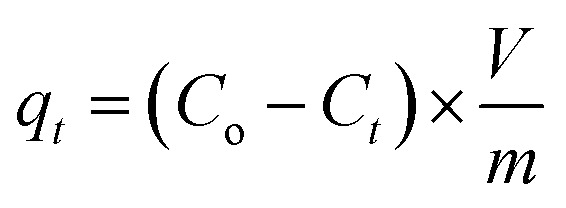


Isotherm fitting and statistical analyses were carried out using a MATLAB environment.

#### 2.9.2. Adsorption of Pb(ii)

Several concentrations of Pb(ii) were prepared from its stock solution (2000 ppm). Adsorption capacities, *q*_Pb_ were determined using eqn (2) by carrying out adsorptions by stirring (rpm 300) CPZA 7 (50 mg) and 50–2000 ppm Pb(ii) solutions (20 mL) at pH 6.0 (adjusted using 0.1 M NaOH and/or 0.1 M HNO_3_) in screw cap glass vials at 298, 313, and 328 K for 6 h. The filtered solutions were analyzed by ICP-MS to determine the concentrations of Pb(ii). Isotherm fitting and statistical analyses were carried out using a MATLAB environment.

The mixtures of CPZA 7 (250 mg), water (88.8 mL), 1 M NaOH (1.2 mL), and 2000 ppm Pb(ii) (10 mL) in 250 mL RB flasks were stirred (300 rpm) at 298, 313, and 328 K. Volume of 1 M NaOH was determined by several pretrial runs to maintain the final pH at 6. The mixture thus became 200 ppm in Pb(ii). The stirring was briefly stopped at several time intervals to collect ≈0.2 mL of the supernatants to determine Pb(ii) concentrations for the kinetic runs. Using eqn (4), adsorption capacities, *q*_*t*_ (mg g^−1^) at various times *t* was determined.

### 2.10. Recycling experiment involving MB and Pb(ii)

#### 2.10.1. Adsorption/desorption of MB

A mixture of CPZA 7 (100 mg) and MB trihydrate (46.7 mg) in water (40 − *x* mL) in a centrifuge tube was stirred, and the pH of this 1000 ppm MB solution was adjusted to 7.25 with the addition of 0.1 M NaOH (*x* mL). After stirring for 6 h at 298 K, the supernatant was analyzed by UV-vis spectroscopy at pH 7 (*vide supra*) for *q*_e_ and *C*_e_.

After centrifugation and removal of the supernatant, the residual resin was washed with water (5 mL). To dislodge MB from the MB-loaded residue, it was stirred twice for 15 min – first with 10 mL 1 M HCl and then with 5 mL 0.1 M HCl. The two supernatants were mixed and analyzed to determine the concentration of the desorbed MB. The regenerated CPZA was washed with water and used to quadruplicate the above cycle.

#### 2.10.2. Adsorption/desorption of Pb(ii)

The sorption/desorption process involving Pb(ii) was carried out as described above for the MB. After stirring a mixture of 100 mg of CPZA 7 and 40 mL of 100 ppm Pb(ii) (pH adjusted to 6.0 with 1 M NaOH) in a centrifuge tube for 6 h at 298 K, the *q*_e_ was determined. CPZA 7 in the centrifuge tube, after decanting off the supernatant, was washed with water (5 mL). For desorption, the Pb-loaded CPZA 7 was stirred twice – first with 10 mL 1 M HNO_3_ for 1 h and then with 5 mL 0.1 M HNO_3_ for 10 min. The two supernatants were mixed and analyzed to determine the concentration of the desorbed Pb(ii). The regenerated CPZA 7 was washed with water and used to quadruplicate the above cycle.

## 3. Results and discussion

### 3.1. Adsorbent (CPZA 7) synthesis

Numerous linear cyclopolymers of industrial importance have been synthesized *via* free radical polymerization of various diallylammonium monomers. The polymer backbone is embedded with cyclic pyrrolidine rings.^16,17^ Copolymerization of maleic acid (MA) with diallylammonium salts having reactivity ratios of almost zero gives alternate copolymers.^18^ Copolymerization precludes the formation of individual homopolymers because of the zero reactivity ratios. To synthesize the cross-linked alternate copolymer, cross-linker 4 was synthesized in excellent yield as outlined in Scheme 1. The alternate copolymerization of monomers 5 (37.4 mol%) and 6 (54.2 mol%) in the presence of cross-linker 6 (8.4 mol%) and TBHP initiator afforded CPZA 7 in 84% yield (Scheme 2). The C=C and = C–H stretching of diallyl motifs in the monomers usually appears at ≈ 1640 cm^−1^ and ≈3080 cm^−1^, respectively.^20,21^ These vibrations are absent in the synthesized resin 7, thereby implying the absence of any residual alkene motifs as it is consumed to form the cyclic rings. Upon treatment with NaOH, pH-responsive CPZA 7 was transformed to its anionic form CAPE 8. The % mol ratio of monomers 5/6/4 incorporated into the resin was approximated to be 37.4 : 54.2 : 8.4, same as the feed ratio; this is expected at such a high conversion. Under the reaction conditions, neither diallyamine salts nor maleic acid can undergo homopolymerization; the polymerization invariably leads to the formation of alternate addition of diallylamine salt and maleic acid.^18^ The high IEC value of 7.65 mmol g^−1^ was attributed to CAPE 8 having four CO_2_^−^ groups in each repeating unit.

### 3.2. Morphology of CPZA 7

BET analysis gave the textural parameters of CPZA 7 as listed in Table 1. For CPZA 7, the surface area was found to be greater than 0.1 m^2^ g^−1^ known for various ionic sorbents.^11,22^

**Table:** Morphology of CPZA 7

BET surface area (m^2^ g^−1^)	Total pore volume (cm^3^ g^−1^)	Pore diameter (nm)
1.62	5.20 × 10^−3^	12.8

### 3.3. TGA of CPZA 7

**Fig. 1:**
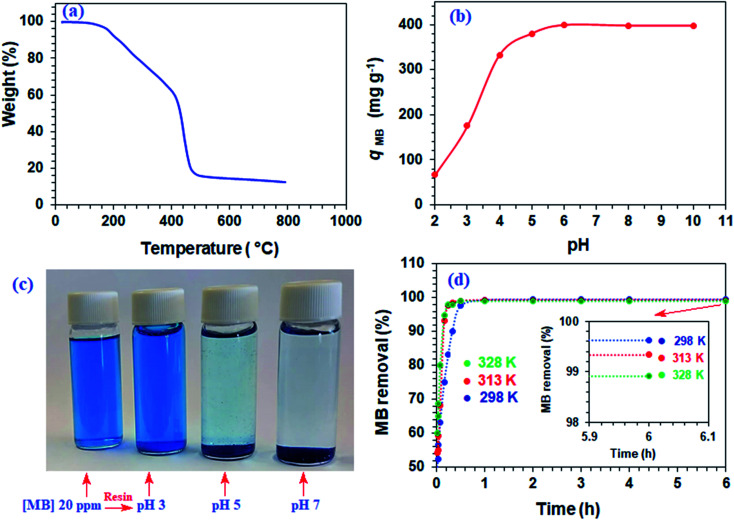
(a) TGA curve of resin CPZA 7; (b) dependency of *q*_MB_ on pH (298 K) [50 mg CPZA 7, 20 mL 1000 ppm MB, 3 h (298 K); (c) photo of CPZA 7 (50 mg) containing 20 mL 20 ppm MB at various pHs (3 h, 298 K); (d) kinetics of percent MB uptake by CPZA 7 at 298, 313, 328 K. [Experimental conditions: CPZA 7 (250 mg), 100 mL 3000 ppm MB, pH 7.25].

The TGA curve for resin 7, shown in Fig. 1a, reveals a weight loss of 7.5% up to 200 °C owing to the moisture loss. A gradual loss of 34% in the 200–415 °C range is attributed to the release of (i) H_2_O during the formation of anhydride units both in the pendant as well as in the backbone and (ii) maleic anhydride units from the pendants.^23,24^ A steep loss of 42% in the 515–800 °C range was accounted by the degradation of the nitrogenated organic fraction along with the removal of maleic acid units from the backbone. The resin was found to be stable up to 200 °C.

### 3.4. Dependency of *q*_e_ on pH

**Scheme 3:**
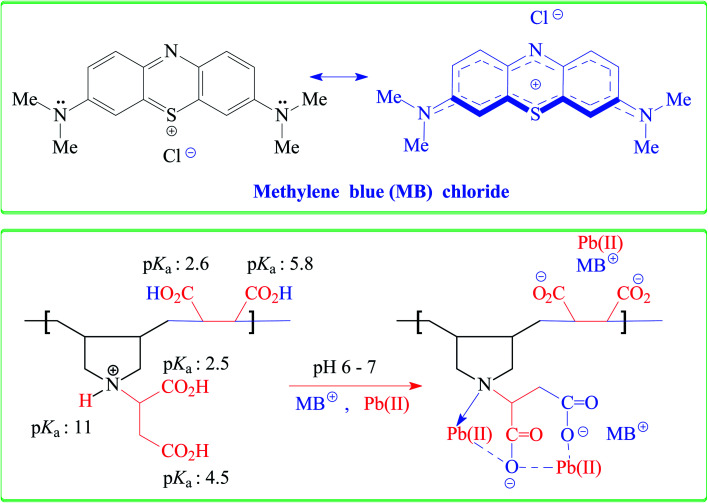
Electrostatic and chemical interactions of CPZA 7 with MB and Pb(ii) at pH 6–7.25.

The *q*_e_ increases with increasing pH and remains constant after pH 7 (Fig. 1b). The succinic acid motifs have two ionizable CO_2_H groups having expected p*K*_a_ of 2.6 and 5.8,^18^ while the p*K*_a_ values of 2.5 and 4.5 could be assigned to the two CO_2_H groups in the residues of aspartic acid residue.^25,26^ In the pH range 6–7.25 used in the current study, the majority of CO_2_H groups is expected to be anionic CO_2_^−^ which can impart electrostatic attraction to entrap cationic MB (Scheme 3). As can be seen in Fig. 1c, the color of MB almost vanishes at pH 7 where the blue-colored resin-MB complex settles down at the bottom.

### 3.5. Adsorption kinetics of MB

**Fig. 2:**
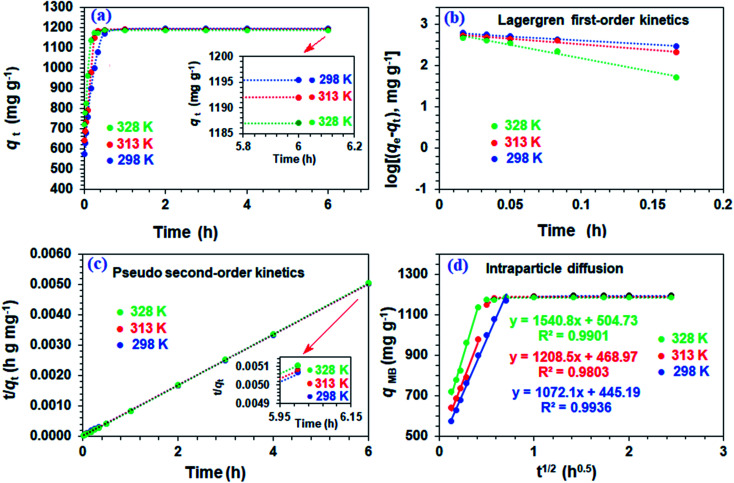
Kinetics of removal of MB by CPZA 7 at ●298, ●313, and ●328 K (pH: 7.25): (a) changes of adsorption capacity, *q*_*t*_ over time; kinetic plots of (b) first-order, (c) second-order, and (d) intraparticle diffusion. [Experimental conditions: resin CPZA 7 (250 mg), 3000 ppm MB (100 mL), pH 7.25].

The resin demonstrated a very fast adsorptive removal of MB with *q*_t_ values attaining the equilibrium *q*_e_ at around ≈15 min (Fig. 2a), whereby 83.3, 97.7, and 98.0% MB is adsorbed from its 3000 ppm solutions at respective temperatures of 298, 313, and 328 K (Fig. 1d). The decrease of *q*_e_ with increasing temperatures reveals the exothermic nature of the adsorption process (inset in Fig. 1d and 2a).

First-order (Fig. 2b), second-order (Fig. 2c), and intraparticle diffusion kinetics (Fig. 2d) were used to analyze the MB adsorption data using eqn (5),^11^(6), and (7), respectively, having corresponding rate constants of *k*_1_, *k*_2_, and *k*_p_.5
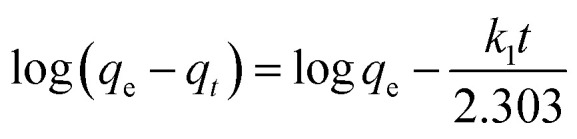
6
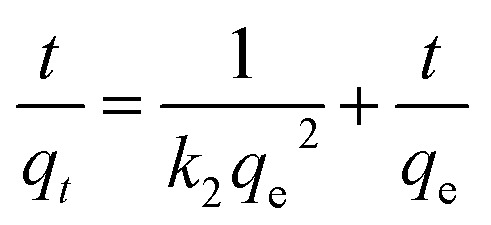

7 *q*_*t*_ = *x*_i_ + *k*_p_*t*^0.5^

The kinetic data fitted the second-order model better as revealed by *R*^2^ and closeness of the *q*_cal_ and *q*_exp_ values, thereby suggesting MB removal as a chemisorption process (Table 2).^27^ The electronic interactions between donor CO_2_^−^ and acceptor cationic aromatic rings may lead to chemisorption.^28,29^ The adsorption processes usually involve film diffusion, followed by intraparticle diffusion and adsorption ⇋ desorption equilibrium (mass action). Intraparticle plots have significant intercepts, *x*_i_ (Fig. 2d), pointing towards the extensive contribution of film diffusion.^30,31^ In the bilinear plots, the initial lines with steeper slopes (*k*_p_) describe the intraparticle diffusion,^32,33^ while the horizontal lines represent (Table 2) the mass action equilibria (Fig. 2d).

Kinetics of the adsorption of MB^a^ on CPZA 7

**Table:** 

Second-order kinetics						
Temp. (K)	*q*_e,exp_ (mg g^−1^)	*k*_2_ (h^−1^ g mg^−1^)	*h* b (h^−1^ mg g^−1^)	*q*_e,calc_ (mg g^−1^)	*R* ^2^	*E*_a_ (kJ mol^−1^)
298	1195	0.02542	36 894	1205	0.9999	33.4
313	1192	0.04429	63 516	1198	1.000	
328	1187	0.08704	123 229	1190	1.000	

a Adsorption of MB (3000 ppm, 100 mL) by CPZA 7 (250 mg) (final pH: ≈ 7.25).

b Initial adsorption rate *h* = *k*_2_*q*_e_^2^.

**Table:** 

First-order kinetics				
Temp. (K)	*q*_e,exp_ (mg g^−1^)	*k*_1_ (h^−1^)	*q*_e,calc_ (mg g^−1^)	*R* ^2^
298	1195	4.915	665.0	0.9986
313	1192	6.345	631.0	0.9832
328	1187	15.21	691.8	0.9816

**Table:** 

Intraparticle diffusion				
Temp. (K)	*k*_p_ (mg g^−1^ h^−1/2^)	*x*_i_ (mg g^−1^)	*R*_i_	*R* ^2^
298	1072	445	0.628	0.9936
313	1209	469	0.607	0.9803
328	1541	505	0.575	0.9901

Eqn (8) was used to calculate the initial adsorption factor (*R*_i_).8
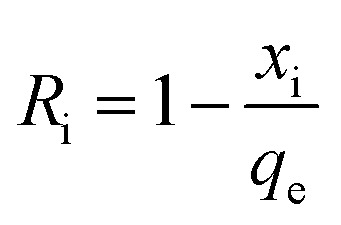


Using the intercept (*x*_i_), which is the instantaneous adsorption at time zero, and *q*_e_, exp values from Table 2, the *R*_i_ values were calculated to be 0.628, 0.607, and 0.575 at respective temperatures of 298, 313 and 328 K, implying the corresponding instantaneous removal of 37.2, 39.3 and 42.5%. The increase in temperature increases the *x*_i_ values, indicating the decrease in resistance to external diffusion.^33^

### 3.6. Infrared spectra

**Fig. 3:**
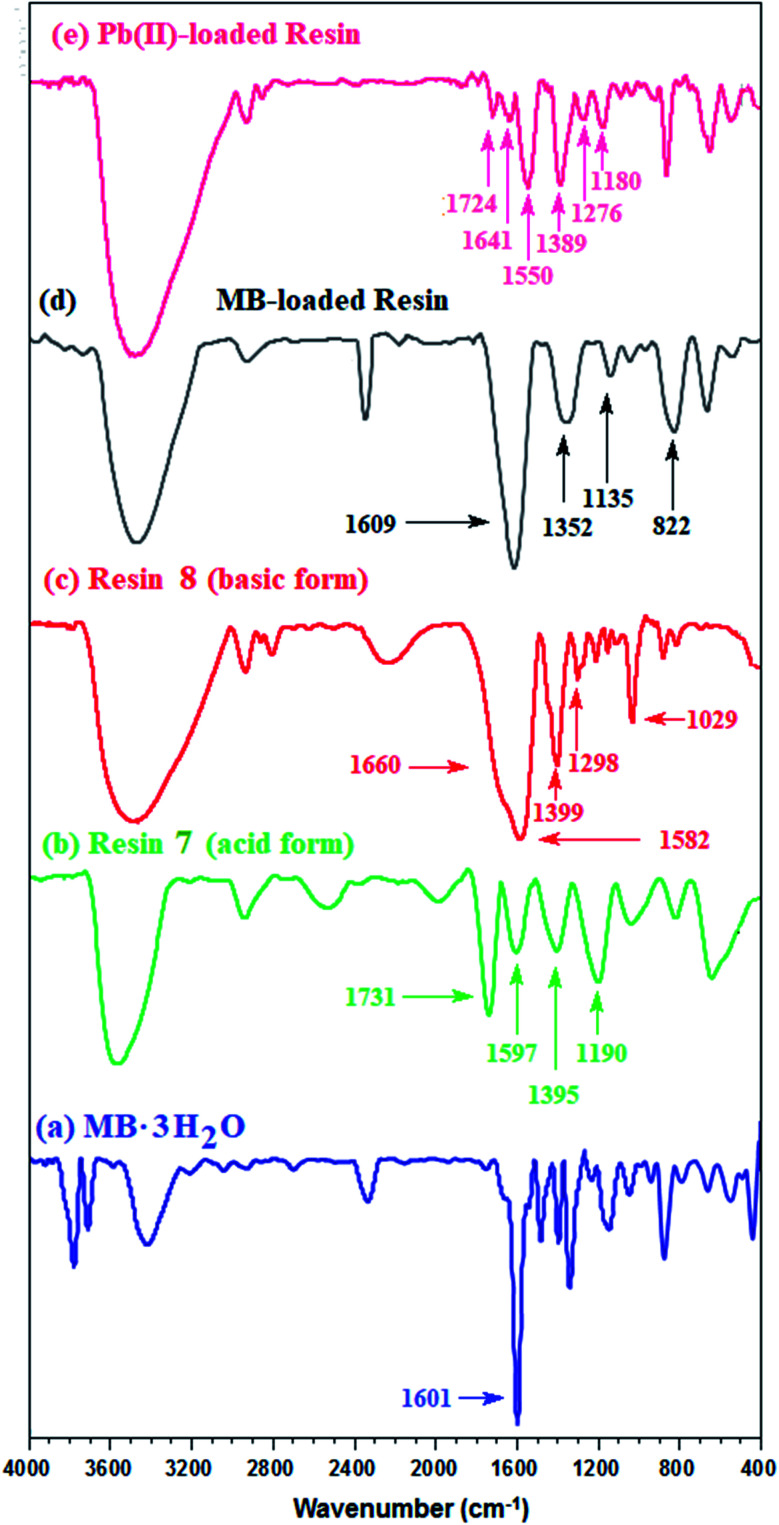
IR spectra of (a) MB + trihydrate; (b) CPZA 7; (c) CAPE 8; resin loaded (d) with MB and (e) Pb(ii).

For the IR spectrum, the MB-loaded resin, collected from the kinetic experiments, was washed and dried under vacuum. The band for the C=O stretching of COOH in CPZA 7A appeared at 1731 cm^−1^, while the adsorption owing to CO_2_^−^ in the zwitterionic motifs in 7 was attributed to the band at 1597 cm^−1^ (Fig. 3b). The bands at 1399 and 1582 cm^−1^ were assigned to the CO_2_^−^ vibrations in the anionic resin 8 (Fig. 3c).^34^ A band at 1601 cm^−1^ was attributed to the ring vibrations of MB·3H_2_O (Fig. 3a).^35^ The considerable shift of C=O band to 1609 cm^−1^ (Fig. 3d) away from that of COOH at 1731 cm^−1^ (Fig. 3b) confirms the CO_2_^−^/MB interactions.

### 3.7. MB adsorption: energy of activation energy (*E*_a_)

**Fig. 4:**
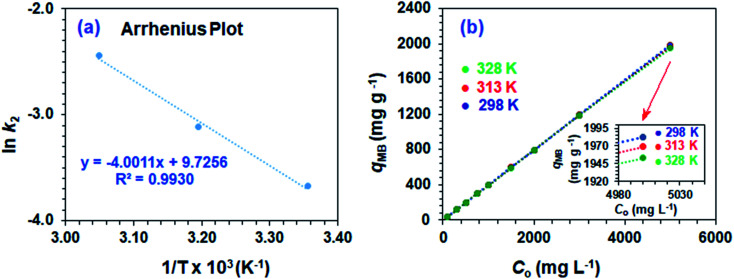
(a) Arrhenius plot and (b) dependency of *q*_e_ of CPZA 7 on the initial MB concentrations (*C*_o_) at 298, 313 and 328 K.

Using second-order rate constants *k*_2_ (Table 2) and the Arrhenius (eqn (9)), the *E*_a_ was found to be 33.4 kJ mol^−1^ (Fig. 4a); the relatively large value implies a chemisorption process.9
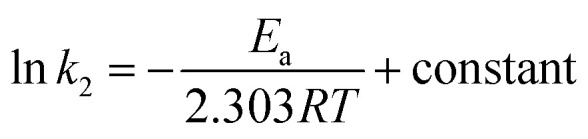


### 3.8. MB adsorption isotherms

A set of *q*_e_ and *C*_e_ using various concentrations (*C*_o_ in the range 100–5000 ppm) of MB at three different temperatures was determined to construct adsorption isotherms. Fig. 4b displays the *q*_e_*versus C*_o_ plots. Using the *q*_e_ and *C*_e_ values at 298 K, linear Temkin, Langmuir, Dubinin–Radushkevich, and Freundlich, isotherms^36^ were constructed (not displayed here) and found to have respective *R*^2^ 0.8865, 0.9995, 0.6965, and 0.9672. The linear Langmuir isotherm thus showed the best fitting with a staggering *q*_m_ value of 2632 mg g^−1^.

The *q*_e_ and *C*_e_ values were used to fit into the nonlinear Langmuir isotherm as per eqn (10) to extract thermodynamic parameters. The units of *q*_e_ and *q*_max_ are in mg g^−1^, while those of *C*_e_ and the Langmuir equilibrium constant *K*_L_ are in mg L^−1^ and L mg^−1^, respectively. Using eqn (11), the equilibrium constant *K*_L_ was converted^37–39^ to 
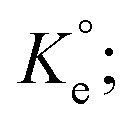
 in Van't Hoff eqn (12), 
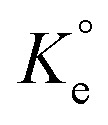
 must be dimensionless. 
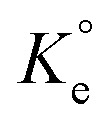
 in eqn (12) becomes unitless by assuming the coefficient of activity, *γ* as dimensionless 1, [MB]° as 1 mol L^−1^ for the standard MB concentration, the molar mass (*M*_w_) in g mol^−1^ of MB as 319.85, and by changing the unit of *K*_L_ from L mg^−1^ to L g^−1^ by multiplying with 1000.10
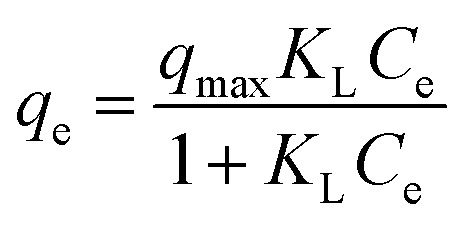
11
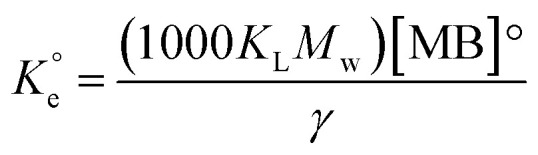
12
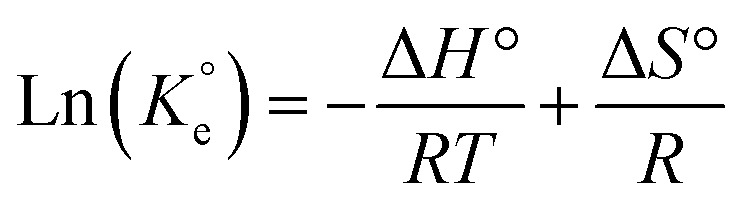


**Fig. 5:**
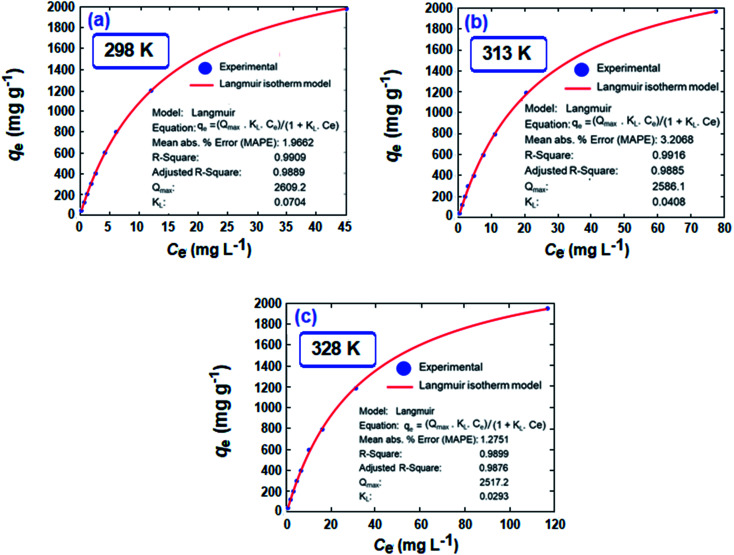
The removal of MB by CPZA 7: nonlinear Langmuir isotherms at (a) 298 K, (b) 313 K and (c) 328 K. [Resin (50 mg), 20 mL MB having *C*_o_ of 100, 300, 500, 750, 1000, 1500, 2000, 3000, and 5000 ppm, final pH: 7.25].

Nonlinear isotherms are displayed in Fig. 5. The *R*_Adj_^2^ values were highly satisfactory; the large *q*_m_ value of 2609, 2586, and 2517 mg g^−1^ at respective temperatures of 298, 313, and 328 K does indeed make CPZA 7 a super-adsorbent (Fig. 5).

**Fig. 6:**
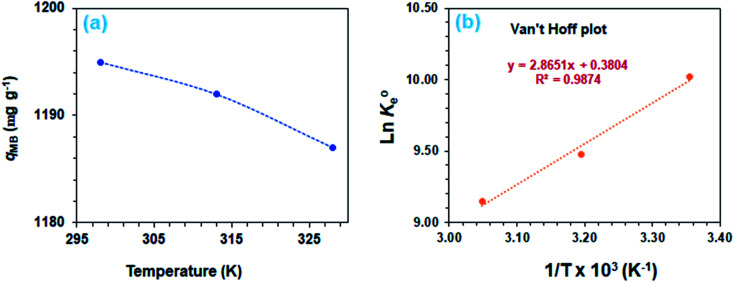
(a) Dependency of MB adsorption capacity, *q*_e_ on temperature [50 mg CPZA 7, 20 mL 3000 ppm MB (*C*_o_), (pH 7.25)] and (b) Van't Hoff plot.

The adsorption process is favorable, as indicated by the relatively large negative values of Δ*G*° (Table 3). The decrease in *q*_MB_ with increasing temperatures makes the adsorption an exothermic process (Fig. 6a). The Δ*H*°, Δ*S*°, and Δ*G*° 
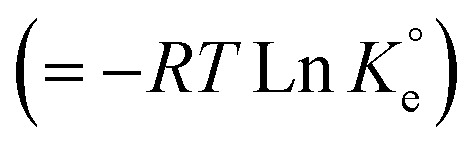
 values, extracted from the Van't Hoff plot (Fig. 6b), are included in Table 3.

**Table:** Thermodynamic parameters for the adsorption MB by CPZA 7

Temp. (K)	*K*_L_ a (L mg^−1^)	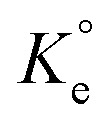 b (dimensionless)	Δ*G*°^c^ (kJ mol^−1^)	Δ*H*^o^ (kJ mol^−1^)	Δ*S*^o^ (J mol^−1^ K^−1^)	*R* ^2^
298	0.0704	22 517	(−) 22.87	(−) 23.8	(+)3.16	0.9874
313	0.0408	13 050	(−) 22.83			
328	0.0293	9372	(−) 22.78			

a From nonlinear Langmuir isotherms.

b Using eqn (11).

c
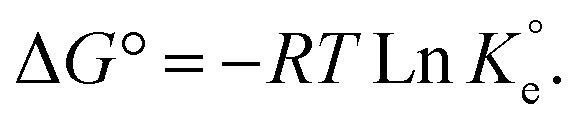

### 3.9. Adsorption of MB in the presence of NaCl

**Fig. 7:**
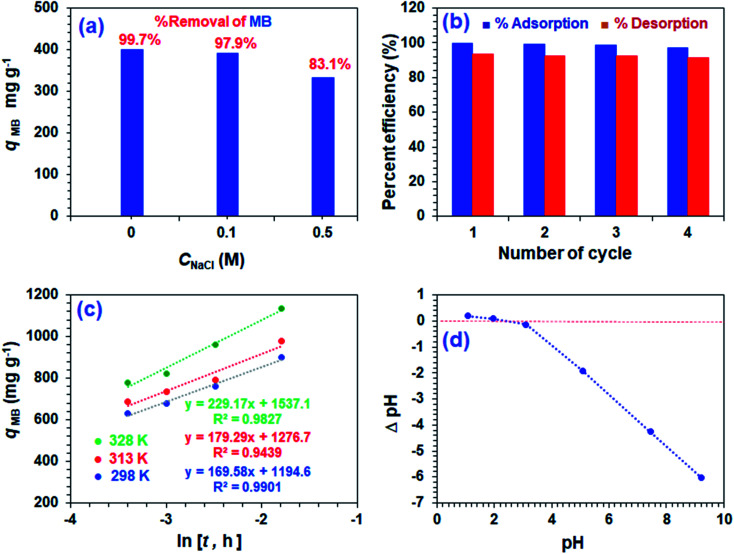
(a) MB adsorption capacity, *q*_e_ and percent removal in the presence of NaCl. [CPZA 7: (50 mg), 1000 ppm MB solution (20 mL)]; (b) percent efficiencies of adsorption/desorption over four cycles. [CPZA 7 (100 mg), 1000 ppm MB (40 mL), pH 7.25]; (c) plot of Elovich kinetic model [250 mg CPZA 7, 100 mL 3000 ppm MB, pH 7.25]; (d) determination of pH of point of zero charge.

Fig. 7a displays the effect of NaCl on the adsorptive MB removal from its 1000 ppm solutions. The industrial effluents containing NaCl may affect the efficacy of the adsorption of MB. It is gratifying to note that the respective percent removal in 0, 0.1, and 0.5 M NaCl was found to be 99.7, 97.9, and 83.1%, thereby confirming no significant adverse effects on the MB removal process by CPZA 7.

The presence of 2300 ppm Na^+^ (0.1 M) and 11 500 ppm Na^+^ (0.5 M) ions are unable to compete effectively with the 1000 ppm of cationic MB for adsorption onto the resin. Thus electronic interactions between the electron-donor CO_2_^−^ ligands and electron-acceptor cationic aromatic rings of MB constituting a chemisorption process are thermodynamically more favorable than the Na^+^–CPZA 7 electrostatic attractions.^28,29^

### 3.10. Sorbent recycling

For the regeneration and reuse in several cycles, the CPZA 7 has achieved excellent efficacies of ≈92–99% (Fig. 7b). The presence of numerous CO_2_H, NH^+^ makes it a pH-responsive resin, thereby providing the latitude of 7 ⇋ 8 equilibrations in the presence of HCl or NaOH. The absence of labile motifs like ester, amide, *etc.*, makes it a chemically robust resin.

### 3.11. Mechanism of adsorption

The kinetic adsorption data are fitted well with the Elovich model (eqn (13)), thereby suggesting the process as chemisorption (Fig. 7c).^40^ The *α* and *β* values of 1.94 × 10^5^ mg g^−1^ h^−1^ and 0.00590 g mg^−1^ at 298 K, respectively, indicate the irreversible nature of the adsorption process because of a very high rate of adsorption (*α*) and low rate of desorption (*β*).^33^ While endothermic adsorption suggests a chemisorption process, the exothermic Δ*H*° of −23.8 kJ mol^−1^ (Table 3) and its magnitude may suggest the physi- and chemi-sorption process.^41^ The Δ*G*° value of −22.8 kJ mol^−1^ suggests the process may involve both monolayer adsorption (chemisorption) and multilayer adsorption (physisorption).^42^ Formation of multilayer dye molecule may account for the ultra-high adsorption capacity, *q*_MB._^43^13
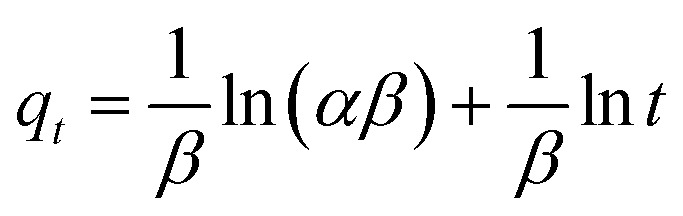


The pH drift method^44^ to determine pH at the point of zero charge (pH PZC) utilized a series of 0.1 M NaNO_3_ (10 mL) solutions in CO_2_-free deionized water at various initial pH values adjusted using 0.1 M NaOH or 0.1 M HCl. After adding CPZA 7 (50 mg) and stirring at 298 K for 24 h, the final pHs were measured. The difference of initial and final pH, *i.e.*, ΔpH plotted against the initial pH plot, revealed a pH value of 2.4 as the pH PZC, representing the point where ΔpH equals zero (Fig. 7d). The pH of 7.25 used for the MB removal, being >2.4, would therefore be negatively charged to facilitate chemical and/or physical adsorption of cationic MB. The adsorbed MB cannot be dislodged from the loaded resin by washing with water or ethanol, suggesting that MB is attached to the resin with a stronger force imparted by chemisorption. The desorption of the cationic dye MB bye HCl, which imparts cationic charges on the resin, is associated with the electrostatic repulsion among the positively charged species.

### 3.12. Comparative *q*_m_ for MB of some recent adsorbents

The *q*_m_ values of some recent sorbents used for MB removal are compared with that of the current resin in Table 4, which reveals the notable efficacy of the current resin CPZA 7. The synthesis of current resin can be scaled up easily using inexpensive starting materials, which makes it an attractive sorbent for the mitigation of MB in industrial wastewater.

**Table:** Adsorption maximum (*q*_m_) of MB by some recent resins compared to CPZA 7

Adsorbents	*q*_m_ (mg g^−1^)	Ref.
Nickel-based MOF	694	45
Cellulosic olive stones	588	46
Nickel alginate/graphene oxide aerogel	537	47
Polymeric multi-layered alginate-based	522	48
Magnetic nano-hybrid	714	49
Graphene functionalized with polyamine	741	50
Graphene/β-cyclodextrin	1134	51
Inorganic–organic hybrid nanowire networks	1188	52
Attapulgite/chitosan	1873	53
Hydrogel based on poly(acrylic acid)	2100	54
Resin containing aminocarboxylate and maleic acid	2101	39
Titanate nanosheets	3937	43
Aminophosphonate/succinate resin	2445	55
Resin with aspartate/succinate motifs	2609	(Current work)

### 3.13. Removal of anionic dyes (methyl orange and Eriochrome black T)

**Scheme 4:**
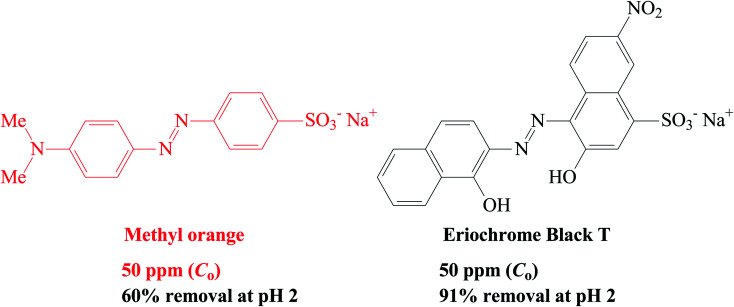
Removal of anionic dyes methyl orange and Eriochrome black T by sorbent 7.

CPZA 7 has been very effective in the trapping of cationic dye MB. The efficacy of 7 in removal of anionic dyes like methyl orange (MO) and Erichrome black T (EBT) has also been examined briefly (Scheme 4). A mixture of each dye (50 ppm, 20 mL) and CPZA 7 (50 mg) was stirred for 6 h at 298 K. Analyses of the supernatants by UV-vis spectroscopy (after adjusting to pH 5.11 for MO and 5.0 for EBT) revealed the removal of 60, 15 and 9.7% MO and 91, 71 and 57% EBT at pH 2, 4, and 6, respectively. The blank MO solutions in the range 1–3 ppm at pH 5.11 and EBT solutions in the range 3–7 ppm at pH 5.0 were used to construct the calibration curves (*λ*_max_ 464 nm for MO and 544 nm for EBT). Decreasing pH thus increases the adsorptive removal of the dyes. At pH 2, all the CO_2_^−^ in CPZA 7 are expected to be protonated to CO_2_H, thereby allowing the cationic NH^+^ motifs in the sorbent to impart electrostatic interactions to bind the anionic SO_3_^−^ groups. Note that SO_3_H groups having a p*K*_a_ of −2.1 is expected to be dissociated to SO_3_^−^ even at pH 2. Some recent works described the removal of EBT using zinc oxide nanoparticles^56^ and magnetic NiFe_2_O_4_ nanoparticles,^57^ achieving the best removal of EBT at about 87 and 91%, respectively.

### 3.14. Adsorption of Pb(ii)

#### 3.14.1. Kinetics runs

After 1 min at 298, 313, and 328 K, 91.6, 94.0, and 95.3% of Pb(ii), respectively, were removed from its 200 ppm solutions (Fig. S1a†). After the very fast adsorption, the equilibrium values for the percent removal and *q*_Pb(II)_ were attained very quickly (Fig. S2a†). The endothermic nature of the adsorption process is established as the *q*_e_ increases with the increase of temperature. Adsorption data fitted well with all three kinetic models (eqn (1)–(3)) as revealed by the kinetic plots in Fig. S1b–d;† however, the closeness of the *q*_cal_ and *q*_exp_ makes the process second-order chemisorption (Table S1†).^27^

Film diffusion dominates the adsorption process as revealed by large intercepts (*x*_i_) (*i.e.*, adsorptions at time zero) values of 72.0, 73.5, and 74.5 mg g^−1^ at the respective temperatures of 298, 313, and 328 K (Fig. S1d†). Using *x*_i_ and *q*_e_, exp from Table S1,† the *R*_i_ values as calculated using eqn (4) were found to be 0.094, 0.0755, and 0.0688 at the respective temperatures of 298, 313, and 328 K, with the corresponding instantaneous adsorption, thus accounting for 90.6, 92.5 and 93%. The resistance to the external diffusion thus decreases with the increase of temperatures, as suggested by increasing *x*_i_ values.^33^ The kinetic data for Pb(ii) adsorption fitted well with the Elovich model (eqn (13)), thereby suggesting the process as chemisorption (Fig. S2b†).^40^ The high adsorption rate (*α*) of ≈1.08 × 10^33^ mg g^−1^ h^−1^ along with the slower rate of desorption (*β*) of ≈0.985 g mg^−1^, point towards the irreversible nature of the chemisorption.^27^

#### 3.14.2. *E*_a_ associated with the Pb(ii) adsorption

The rate constants, *k*_2_ (Table S1†), were used to construct the Arrhenius plot [using eq (5)] (Fig. S3a†). The *E*_a_ was calculated to be 40.7 kJ mol^−1^. Very fast uptake was demonstrated by removing 97% Pb(ii) in 3 min (Fig. S1a†).

#### 3.14.3. Pb(ii) isotherms

Like in the adsorption experiments involving MB, *q*_e_ and *C*_e_ values for a series of Pb(ii) solutions in the concentration range (*C*_o_), 50–2000 ppm were determined at three different temperatures to construct the isotherms. Adsorption capacities, *q*_e_ were found to increase with the increase of *C*_o_ (Fig. S3b†). The data fitted into several linear isotherms (*vide supra*) revealed the best fitting for the Langmuir (*R*^2^ = 0.9976), while the Dubinin–Radushkevich, Temkin, and Freundlich isotherms have respective lower *R*^2^ of 0.7778, 0.9306, and 0.9550 at 298 K.

As discussed in the case of MB (*vide supra*), the adsorption data at the three different temperatures were fitted into Langmuir's nonlinear isotherm (Fig. S4†) to find out the values of *K*_L_ and dimensionless equilibrium constants 
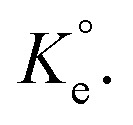
 To do so, [MB]° in eqn (11) was replaced with [Pb]°, and *M*_W_ for Pb was taken as 207.2 g mol^−1^. The *R*_Adj_^2^ values indicated the excellent fitting. The *q*_m_ values were found to be 872.6, 914.9, and 948.4 mg g^−1^ at the respective temperatures of 298, 313, and 328 K (Fig. S4†).

The plot as per Van't Hoff eqn (12) (Fig. S5b†) using 
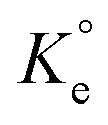
 values revealed the endothermic nature of Pb(ii) adsorption with a relatively high Δ*H*°[(+) 27.8 kJ mol^−1^] (Table S2†), which indicates the process as chemisorption.^58^ The Δ*G*° values, being negative, favors the adsorption process. As a consequence of the endothermic process, an increase in temperatures leads to higher adsorption capacities, *q*_Pb_ (Fig. S5a†).

#### 3.14.4. Remediation of industrial wastewater

The results of the treatment of industrial wastewater with CAPE 8 are given in Table 5. The concentrations of most of the priority toxic metals like Cr, Pb, Hg were decreased to below ppb levels. The resin was also influential in decontaminating the wastewater spiked with 10 000 ppb Pb(ii), which was reduced to 147 ppb (*i.e.*, 98.5% removal). Moreover, the concentration of other metal contaminants was also reduced. It is worth mentioning that the treatment of a mixture containing 10 000 ppb each of MB and Pb(ii) with PZCA 7 led to the more effective removal of MB (97%) than Pb(ii) (69.6%).

**Table:** Comparison of metal concentrations in wastewater sample before and after the treatment with CAPE 8

Metal	Original wastewater (μg L^−1^)	After treatment with CAPE 8^a^ (μg L^−1^)	
		Original wastewater	Original wastewater spiked with Pb^2+^ (10 000 μg L^−1^)
Al	25.4	5.31	6.81
Zn	26.9	0.72	0.19
Fe	32.5	8.41	12.5
Cr	2.89	0.59	1.07
Ni	22.3	2.11	2.33
Co	1.48	0.32	0.34
As	0.16	0.06	0.08
Cu	25.7	4.83	5.67
Sn	3.56	1.27	1.45
Cd	0.63	0.29	0.32
Sn	5.01	1.02	1.12
Hg	1.58	0.45	0.32
Mn	10.4	0.24	0.07
Pb	3.78	0.98	147
			Solution^b^ containing 10 000 μg L^−1^ each of MB and Pb(ii) stirred with PZCA 7
MB	10 000		310
Pb	10 000		3036

a Wastewater (20 mL, pH 5.5) stirred with 50 mg CAPE 8 for 6 h at 298 K.

b 20 mL solution stirred with 50 mg of CPZA 7 (pH 6.0) at 298 K for 6 h.

#### 3.14.5. Pb(ii) desorption

Adsorption and desorption of Pb(ii) have been carried out to examine the effectiveness of CPZA 7 for its potential use in industrial applications for wastewater treatment. The sorption and desorption efficiencies for four cycles were found to be stable in the ranges 94–99% and 92–94%, respectively.

### 3.15. SEM and EDX analysis

**Fig. 8:**
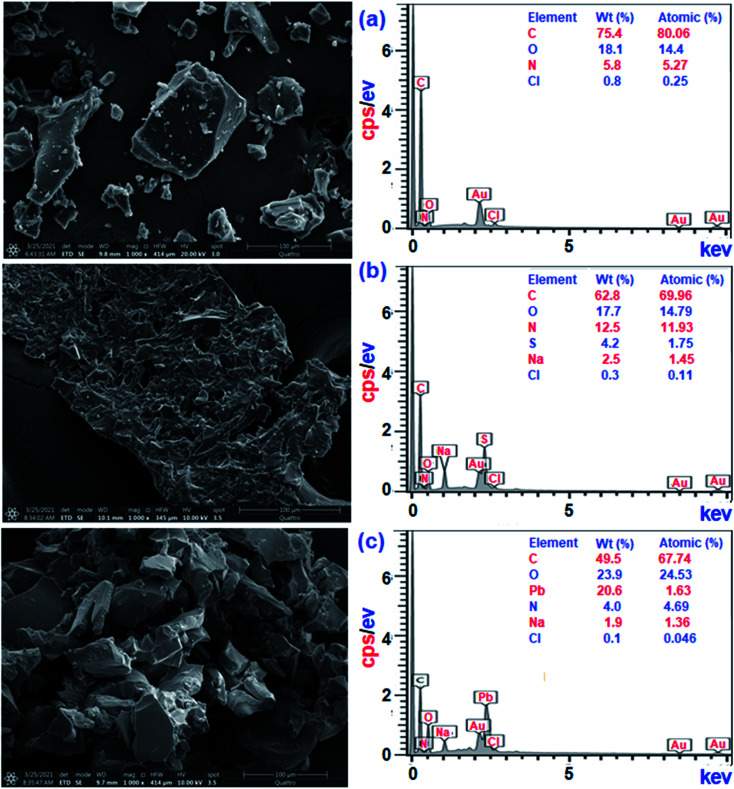
SEM and EDX analysis for CPZA 7 (a) pure, and loaded with (b) MB & (c) Pb(ii).

Treatment of CPZA 7 (100 mg) at 298 K with 40 mL of 1000 ppm MB (pH 7.25, 2 h) and 40 mL of 100 ppm Pb(ii) (pH 6.0, 1 h) afforded resins loaded with MB and Pb(ii), respectively. The sputter-coated nascent and loaded CPZA 7 with a gold film were scanned to obtain their SEM images and EDX spectra, shown in Fig. 8. Upon adsorption, the morphology of the loaded resins was changed (Fig. 8b and c) compared to unloaded CPZA 7 (Fig. 8a). The presence of S and Pb confirms their adsorptions onto the resin. Noted that the C=O vibrations for the Pb(ii)-loaded resin are present as minor bands at 1724 and 1641 cm^−1^, while the strong band shifted to 1550 cm^−1^ indicates the formation of Pb(ii) complex with CO_2_^−^ groups (Fig. 3e).^59^

### 3.16. Pb(ii) adsorption: comparative *q*_m_ of several recent sorbents

The *q*_m_ values of some recent sorbents used for Pb(ii) removal are compared with that of the current resin in Table 6, which reveals the excellent efficacy of CPZA 7.

**Table:** A comparison of maximum Pb(ii) adsorption capacities (*q*_m_) of the CPZA 7 and some similar recent adsorbents in literature

Adsorbents	*q*_m_ (mg g^−1^)	Ref.
Starch-based ZnO nanocomposite	256	60
Chitosan/lignosulfonate adsorbent	525	61
Polypyrrole-iron oxide-seaweed nanocomposite	333	62
Polyvinyl alcohol phosphate ester super-adsorbents	559	63
Bentonite modified chitosan–cellulose	256	64
Treated sodium alginate	221	65
Naphthalene sulfonic acid doped polyaniline nickel composite nanotubes	415	66
Nitrogen-doped carboxylated porous carbon	721	67
Amido-amine derivative of alginic acid	556	68
Resin with aspartate/succinate motifs	873	(Current work)

## 4. Conclusions

Monomer 5 containing residue of aspartic acid [(CH_2_=CHCH_2_)_2_NH^+^CH(CO_2_H)CH_2_CO_2_H Cl^−^] underwent TBHP-initiated alternate copolymerization with maleic acid 6 in the presence of cross-linker tetraallylhexane-1,6-diamine dihydrochloride [(CH_2_=CHCH_2_)_2_NH^+^(CH_2_)_6_NH^+^ (CH_2_=CHCH_2_)_2_ 2Cl^−^] 4 to afford a new pH-responsive cross-linked chelating ion-exchange resin CPZA 7 in excellent yield (87%). The resin containing an abundant CO_2_^−^ and chelating motifs of succinate (–CHCO_2_^−^–CHCO_2_^−^) and R_2_NCHCO_2_^−^ has been turned out to be a super-adsorbent for the removal of cationic MB and Pb(ii) with *q*_max_ of 2609 and 872.6 mg g^−1^, respectively, at 298 K. Both MB and Pb(ii)-uptake was found to be very fast; obeying second-order kinetics, the resin accounted for 97.7% removal of MB within 15 min at 313 K and 94% Pb(ii) removal within 1 min. The removal data for MB and Pb(ii) fitted Elovich kinetic model, thereby supporting a chemisorption process. The thermodynamic parameter Δ*H*°, extracted from nonlinear Langmuir adsorption isotherms, was found to be (−) 23.8 kJ mol^−1^ (exothermic) and (+) 27.8 kJ mol^−1^ (endothermic) for the respective adsorptions of MB and Pb(ii). The adsorption efficiency of CPZA 7 remained stable for over 4 cycles of adsorption/desorption involving MB and Pb(ii).

CPZA 7 was also found to trap anionic dyes; it removed 60% MO and 91% EBT from their 50 ppm solutions at pH 2.

CPZA 7 performed much better than many recently reported sorbents to remove MB and Pb(ii). The presence of NaCl (0.1–0.5 M) did not have any significant adverse effect on the removal of MB. CPZA 7 was found to be effective in reducing priority metal contaminants (like Cr, Hg, Pb) in industrial wastewater to sub-ppb levels. The resin also performed well for the removal of MB and Pb(ii) simultaneously from their solution. The synthesis of the resin can be easily scaled up from inexpensive starting materials; its outstanding performances pave the way for potential application in wastewater treatment.

## Conflicts of interest

There are no conflicts to declare.

## Supplementary Material

RA-012-D1RA09234K-s001
