# Supplementary material for: A sorbent containing pH-responsive chelating residues of aspartic and maleic acids for mitigation of toxic metal ions, cationic, and anionic dyes
Source: RSC Adv. 2022 Feb 17;12(10):5938–52. doi: 10.1039/d1ra09234k (PMC8981974; doi:10.1039/d1ra09234k)
Supplement: RA-012-D1RA09234K-s001 [file RA-012-D1RA09234K-s001.pdf]

Electronic Supplementary Material (ESI) for RSC Advances.
This journal is © The Royal Society of Chemistry 2022

Supporting Information

A resin containing pH-responsive chelating residues of aspartic and maleic acids for mitigation of toxic metal ions and methylene blue

Shaikh A. Ali*[a,b], Shuaib A. Mubarak[a], Ibrahim Y. Yaagoob[a], Zeeshan Arshad[a], and Mohammad A. J. Mazumder* [a,b]

[a]*Chemistry Department, King Fahd University of Petroleum & Minerals, Dhahran 31261, Saudi Arabia.*

[b]*Interdisciplinary Research Center for Advanced Materials, King Fahd University of Petroleum & Minerals, Dhahran 31261, Saudi Arabia*

Table S1. Kinetics of the adsorption of Pb(II)[a] on CPZA 7.

Temp (K)	$q_{e,\ exp}$ (mg g^{-1})	k_2 (h^{-1}g mg^{-1})	h^b (h^{-1}mg g^{-1})	$q_{e,\ calc}$ (mg g^{-1})	R^2	E_a (kJ mol^{-1})
Second-order						
298	79.49	1.812	11469	79.56	0.9999	40.7
313	79.72	4.184	26610	79.75	1.0000	
328	79.83	8.126	51814	79.85	0.9999	
First-order						
Temp	$q_{e,\ exp}$	k_1		$q_{e,\ calc}$	R^2	
[illegible]	[illegible]	[illegible]		[illegible]		
298	79.49	3.023		6.378	0.9928	
313	79.72	8.546		5.384	0.9980	
328	79.83	9.656		3.877	0.9654	
Intraparticle diffusion						
Temp (K)	k_p mg g^{-1} h$^{-1/2}$	x_i (mg g^{-1})		R_i	R^2	
298	9.090	72.01		0.0941	0.9955	
313	10.41	73.54		0.0775	0.9934	
328	11.64	74.50		0.0668	0.9973	

[a]Adsorption of Pb(II) (200 ppm, 100 mL) by CPZA **7** (250 mg) (final pH: 6.0).

[b]Initial adsorption rate h = $k_2\, q_e^2$.

Table S2. Thermodynamic parameters for the adsorption Pb(II) by CPZA **7**.

Temperature (K)	$K_L{}^a$ (L mg^{-1})	$K_e^{o\,b}$ (unit less)	$\Delta G^{o\,c}$ (kJ mol^{-1})	ΔH^o (kJ mol^{-1})	ΔS^o (J mol^{-1} K^{-1})	R^2
298	0.072	14918	(-) 23.8	(+) 27.8	(+)173	0.9909
313	0.1246	25817	(-) 26.4			
328	0.2012	41689	(-) 29.0			

^{a}From Langmuir nonlinear isotherms. ^{b}using Eq (10). $^c\Delta G^o = -\,RT \ln K_e{}^o$

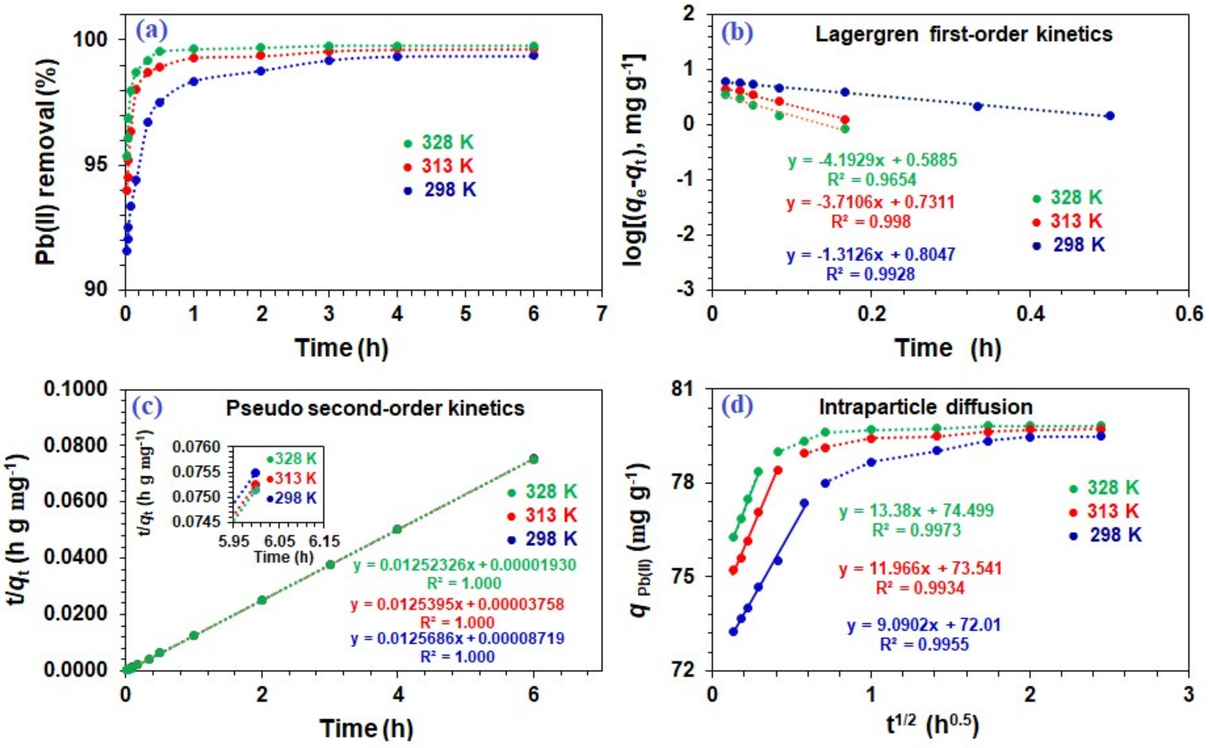

Figure S1. Kinetics of removal of Pb(II) by CPZA **7** at ●298, ●313, and ●328 K: Kinetic plots of (**a**) percent Pb(II) uptake by CPZA **7**; (**b**) First-order, (**c**) Second-order, and (**d**) Intraparticle diffusion. [Experimental conditions: CPZA **7** (250 mg), 200 ppm MB (100 mL), pH 6.0]

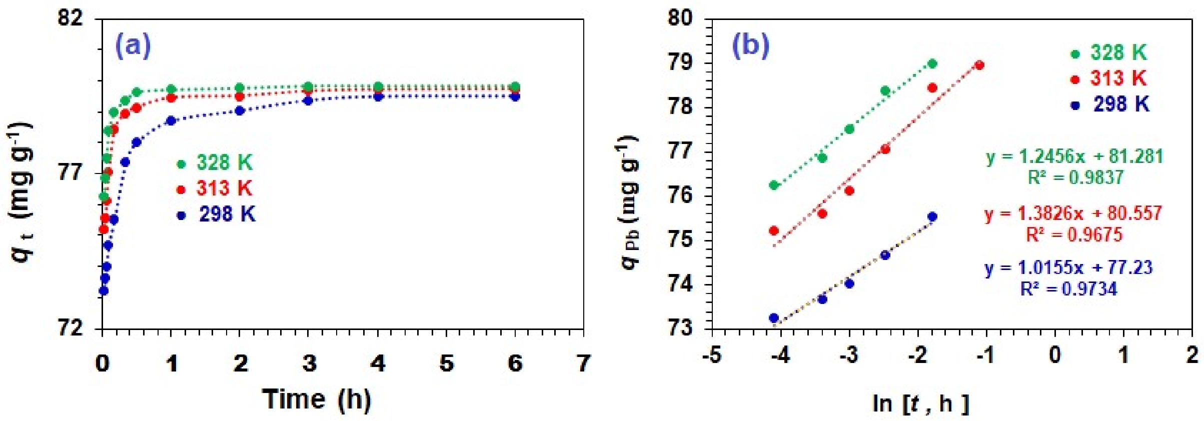

Figure S2. (**a**) Changes of adsorption capacity, q_t of Pb(II) over time; (**b**) Plot of Elovich kinetic model. [Experimental conditions: 250 mg CPZA **7**, 100 mL 200 ppm Pb(II), pH 6.0];

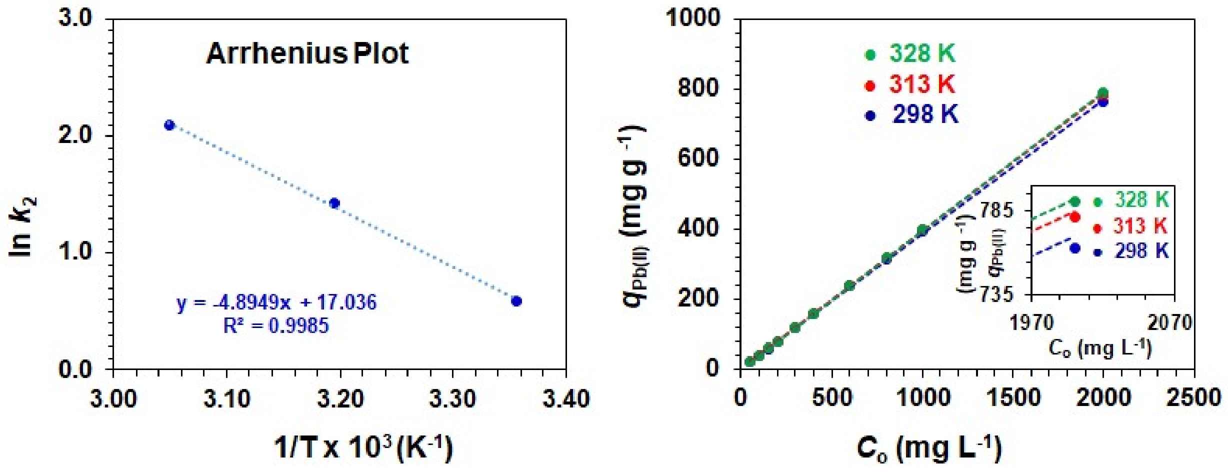

Figure S3. **a**) Arrhenius plot and (**b**) dependency of q_e of CPZA **7** on the initial Pb(II) concentrations (C_o) at 298, 313, and 328 K

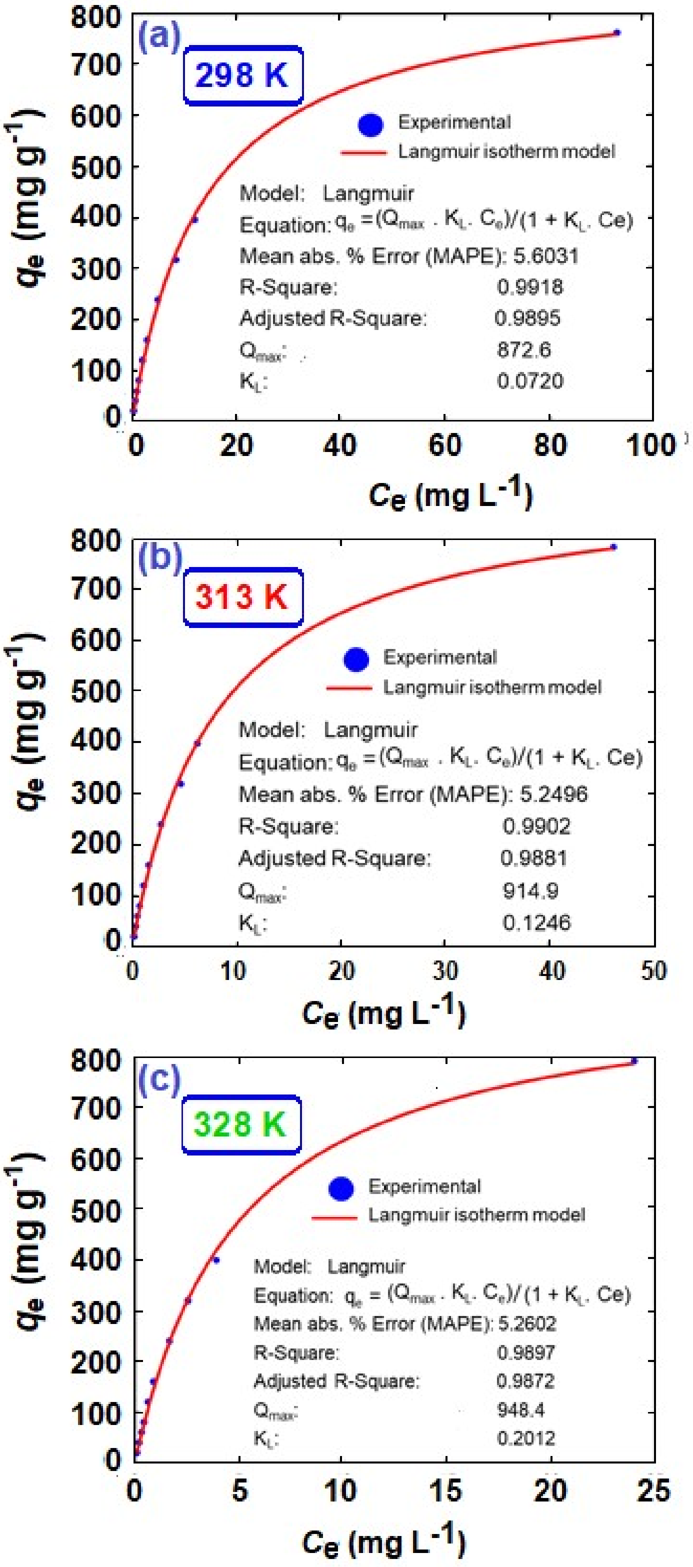

Figure S4. The removal of Pb(II) by CPZA **7:** Nonlinear Langmuir Isotherms at (a) 298 K, (b) 313 K and (c) 328 K. [Resin (50 mg), 20 mL Pb(II) having C_o 50, 100, 150, 200, 300, 400, 600, 800, 1000, and 2000 ppm, final pH: 6.0].

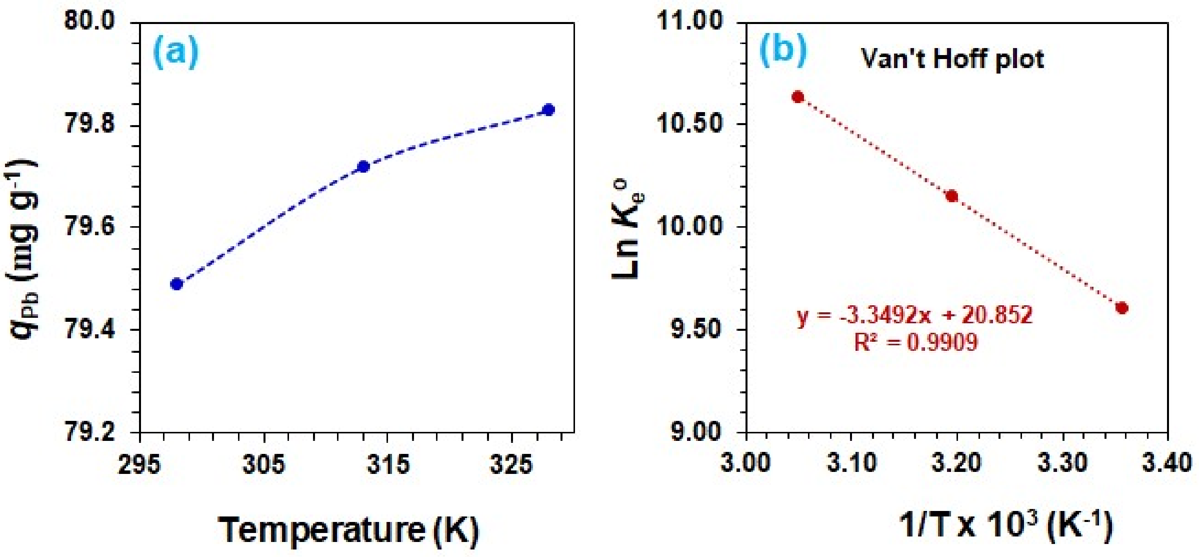

Figure S5. **(a)** Dependency of Pb(II) adsorption capacity, q_e on temperature [50 mg CPZA **7**, 20 mL 200 ppm Pb(II) (C_o), pH 6.0)] and **(b)** Van't Hoff plot.
